# Association of cesarean delivery timing with pelvic floor muscle function and urine incontinence: A propensity score‐matched study

**DOI:** 10.1002/SMMD.20220018

**Published:** 2022-12-27

**Authors:** Yiyao Chen, Chuangchuang Xu, Qimanguli Saiding, Xiaolei Chi, Lei Chu, Xianjing Wang, Xinliang Chen

**Affiliations:** ^1^ Department of Obstetrics and Gynecology International Peace Maternity and Child Health Hospital School of Medicine Shanghai Jiao Tong University Shanghai China; ^2^ Pelvic Floor Clinic Center International Peace Maternity and Child Health Hospital School of Medicine Shanghai Jiao Tong University Shanghai China; ^3^ Shanghai Key Laboratory of Embryo Original Disease Shanghai China

**Keywords:** cesarean delivery, intrapartum cesarean delivery, pelvic floor dysfunction, pelvic floor muscle surface electromyography, propensity score‐matched analysis

## Abstract

Pelvic floor dysfunction is a common gynecological disease that adversely affects women's quality of life and mental health. Delivery is considered a significant independent risk factor for pelvic floor dysfunction. Surface electromyography (sEMG) values for the pelvic floor muscles (PFM) have been shown to differ according to different delivery modes. This study aimed to compare sEMG results between intrapartum and antepartum cesarean delivery (CD), 42–60 days after delivery. Data of women who underwent CD at the International Peace Maternity and Child Health Hospital were collected from September 2021 to December 2021. Myotrac Infiniti System was used to measure the electromyographic activity of PFM after 42–60 days of parturition. Propensity score matching (1:1) was applied to achieve a balance in baseline data between the two groups (intrapartum and antepartum CD). A total of 200 paired cases were selected for statistical analysis. In the propensity score‐matched analysis, there were no statistically significant differences in PFM sEMG between women with antepartum or intrapartum CD (*p* > 0.05 for all). We observed similar results with postpartum urinary incontinence (24 [12.0] vs. 21 [10.5]; adjusted odds ratio (aOR), 1.12 [95% confidence interval (CI) 0.60–2.12]; *p* = 0.717) and stress urinary incontinence (12 [6.0] vs. 14 [7.0]; aOR, 0.80 [95% CI 0.35–1.80]; *p* = 0.596) as outcomes. After excluding participants with intrapartum CD when the cervix was dilated <6 cm, all sEMG of PFM had a comparable level of risk in both the antepartum and intrapartum CD groups. There were no significant differences in sEMG of the PFM and the incidence of urinary incontinence between patients undergoing intrapartum or antepartum CD. Excluding women who underwent intrapartum CD when the cervix was dilated to <6 cm produced little change in results. Thus, different opportunities for CD may not impact the sEMG of the PFM and the incidence of urinary incontinence.

## Graphical abstract

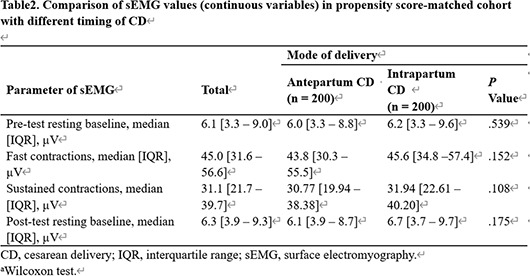

In the present study, patients who underwent the first CD at the International Peace Maternity and Child Health Hospital from September 2021 to December 2021 were included for analyzing the effect of intrapartum CD compared to antepartum CD on the activity of the PFM. The patients were divided into an antepartum CD group and an intrapartum CD group according to delivery mode. The propensity score‐matched cohort included 400 primiparous women who underwent CD, 200 each in the antepartum and intrapartum CD groups. In the propensity score‐matched analysis, there were no statistically significant differences in PFM sEMG between women with antepartum or intrapartum CD.

1

**Key points**

- The developing abnormal surface electromyographic values (sEMG) of PFM and urinary incontinence (UI) after intrapartum and antepartum CD was compared in a propensity‐matched cohort.
- After delivery, the odds of having PFM abnormalities and the incidence of UI did not vary by the timing of CD.
- In the subgroup analysis and sensitivity analysis, the findings remained stable.

## 2 INTRODUCTION

Pelvic floor dysfunction is a series of gynecological diseases caused by damage, degradation, or defects in the pelvic floor support structure with consequent effects on function. The most common clinical symptoms include pelvic organ prolapse, urinary incontinence, and sexual disorders. Pelvic floor dysfunction is also associated with emptying disorders of the bladder, chronic pelvic pain, fecal incontinence, and emptying disorders of the bowel.^1^ Pelvic floor dysfunction is prevalent among women, with 20%–50% of them suffering from pelvic organ prolapse and 2.6%–28.7% from urinary incontinence.^2^, ^3^ The incidence of depressive symptoms in women with pelvic floor dysfunction is > 20%, adversely affecting their quality of life and mental health.^4^, ^5^

Risk factors for pelvic floor dysfunction include age, body mass index, several pregnancies, delivery, hysterectomy, etc.^6^, ^7^ Among the causes of pelvic floor dysfunction, obstetric factors, such as mode of delivery, are regarded as important independent risk factors.^8^ Several researchers have studied the effects of different delivery methods on postpartum pelvic floor function in women. Most of these studies have indicated that vaginal delivery increases the incidence of pelvic floor dysfunction compared to cesarean delivery (CD).^8^, ^9^, ^10^, ^11^ This difference may be related to the degree of levator ani muscle injury caused by the delivery. However, it is still unknown whether intrapartum CD of primiparas aggravates the damage to the pelvic floor muscles (PFM) and decreases their strength. The purpose of this study, which applied sEMG, was to analyze the effect of intrapartum CD compared to antepartum CD on the activity of the PFM.

## 3 METHODS

### 3.1 Study design and data collection

This retrospective cohort study included all female patients who underwent the first CD at the International Peace Maternity and Child Health Hospital, Shanghai, China, from September 2021 to December 2021.

The patient data registry was used to select patients who met the following inclusion criteria: (1) aged between 20 and 45 years, (2) singleton pregnancy with CD, and (3) no lochiorrhea.

The exclusion criteria were as follows: (1) age <20 years or > 45 years; (2) preterm birth; (3) multiple pregnancies; (4) pelvic organ prolapse or urinary tract infection before pregnancy; (5) screening time beyond 42–60 days; (6) rectus abdominis involvement surpassed 20%; (7) cognitive alteration; and (8) missing data.

### 3.2 Exposure

The patients were divided into an antepartum CD group and an intrapartum CD group according to delivery mode. The antepartum CD was defined by an absent trial of labor and none of the following criteria: induction of labor, augmentation of labor, instrument‐assisted labor, shoulder dystocia, vaginal lacerations, CD performed for failure to progress or failed induction, full cervical dilation, or onset of spontaneous labor.

All other CDs that did not meet the definition of antepartum CD were classified as intrapartum. According to Zhang et al., the transition from the latent to the active phase of labor is at approximately 6 cm of cervical dilatation.^12^ Thus, 56 patients who underwent intrapartum CD when the cervix was dilated (≥6 cm) were included in the further analysis (Figure 3).

### 3.3 Outcomes and measurement

A trained gynecologist recorded the electromyographic activity of the PFM at the International Peace Maternity and Child Health Hospital after 42–60 days of parturition. A Myotrac Infiniti System (Montreal, Canada) with endovaginal probes (CACB04, MLD V1, Medlander Medical Instruments Co., Ltd., Nanjing, China) was used for surface electromyography (sEMG). The device acquisition frequency was set to range from 50 to 1000 Hz. All participants received instructions on activating their PFM and avoiding using their gluteal, hip, or abdominal muscles before the sEMG assessment. For parameter measurements, the supine lithotomy position was used.

According to the modified Glazer's protocols and guidelines for the assessment of muscle sEMG,^13^ the measurement procedure of sEMG was divided into the following parts: pretest resting baseline (resting electromyographic activity of PFM 10 s before the measurement), fast contractions (maximum voluntary contraction of the PFM), sustained contractions (five contractions of the PFM held for 10 s), and posttest (electromyographic activity of PFM after resting for 60 s). The sEMG data were standardized using Myotrac Infiniti System in microvolts.

Parameters related to urinary incontinence were obtained from a self‐filled International Consultation on Incontinence Questionnaire‐Urinary Incontinence (ICIQ‐UI) short form. From this, Question 3, “How often did you experience urine leakage?” was used to determine the prevalence of urinary incontinence. Question 6, “When does urine leak?” was used to determine the type of incontinence. The ICIQ‐UI short form has been translated into Chinese and its validity and reliability have been established.^14^

### 3.4 Data collection

Demographic and obstetric data were extracted from the electronic medical record system, including maternal age, body mass index (BMI) before pregnancy, weight gain during pregnancy, infant weight, gestational week, miscarriage history, and complications, including diabetes. These covariates were included because of previously reported differences in pelvic floor dysfunction.^15^ Screening time after delivery was calculated based on the childbirth date and sEMG examination time (Table 1).

**TABLE 1:** Descriptive characteristics of participants before and after propensity score matching

	No. (%)					
	Pre‐matched, mode of delivery			Post‐matched, mode of delivery		
Characteristics	Antepartum CD (*n* = 493)	Intrapartum CD (*n* = 214)	SMD^a^	Antepartum CD (*n* = 200)	Intrapartum CD (*n* = 200)	SMD^a^
Baseline characteristics						
Age, mean (SD), y	31.98 (3.7)	31.3 (3.0)	0.188	31.5 (3.6)	31.4 (3.1)	0.014
Prepregnancy BMI, mean (SD), kg/m^2^	21.84 (3.3)	21.7 (2.6)	0.063	21.5 (3.1)	21.8 (2.6)	0.077
Weight gain in pregnancy, mean (SD), kg	13.68 (5.0)	13.52 (4.8)	0.032	13.7 (4.6)	13.6 (4.9)	0.031
Infant weight, mean (SD), g	3257.0 (514.9)	3384.9 (415.9)	0.273	3380.2 (489.5)	3372.3 (419.9)	0.017
Gestation week, media [IQR]	39 [38–39]	39 [39–40]	0.556	39 [39–40]	39 [39–40]	0.031
Miscarriage history	170 (34.5)	68 (31.8)	0.058	61 (30.5)	64 (32.0)	0.032
Complications						
Hypertensive disorders	83 (16.8)	19 (8.9)	0.239	16 (8.0)	18 (9.0)	0.036
Diabetes (gestational/pregestational)	112 (22.7)	45 (21.0)	0.041	39 (19.5)	41 (20.5)	0.025
Others	77 (15.6)	25 (11.7)	0.115	20 (10.0)	25 (12.5)	0.079
Screening characteristics						
Screening time after delivery, mean (SD), d	47.15 (3.46)	47.30 (3.40)	NA	47.05 (3.48)	47.24 (3.36)	NA
Abdominal involvement ratio, media [IQR], %	6.77 [2.59–11.82]	7.42 [3.14–11.61]	NA	6.72 [1.88–11.28]	7.52 [3.19–11.66]	NA

Abbreviations: CD, cesarean delivery; IQR, interquartile range; SD, Standard deviation; SMD, standardized mean difference.

^a^ SMD value lower than 0.1 was considered a good balance.

### 3.5 Statistical analysis

Propensity‐matched scoring was applied to balance baseline data between the exposed and control groups (i.e., minimal confounding). A multivariate logistic regression model was used to calculate the propensity score with the timing of CD as the dependent variable. Age, prepregnancy BMI, weight gain during pregnancy, complications (hypertension, diabetes, and others), gestational week, infant weight, and miscarriage history were the covariates. The caliper width was set to 0.02, and the matching procedure was completed with a 1:1 ratio and no replacement (greedy matching method). The absolute standardized mean difference (SMD) was used to estimate the balance of the baseline data between the two groups before and after matching. An SMD value lower than 0.1 was considered a good balance.

In the matched cohort, the Wilcoxon test estimated the differences in the distribution of sEMG between the two groups as a continuous variable. Then, by transforming each sEMG value into a binary variable according to the median value, odds risks (ORs) of abnormal level values were estimated for women with antepartum CD and intrapartum CD. Previous studies have reported that the timing of postpartum screening and abdominal involvement may be potential contributors to the outcomes. Considering that these factors could not be classified as baseline characteristics, we included them in a multivariate regression model to obtain the adjusted ORs in the post‐matched cohort.

Two sensitivity analyses were conducted. First, considering that prepregnancy BMI, weight gain during pregnancy, and infant weight are related to postpartum PFM function and that mode of delivery varies according to age, we stratified participants according to age (<35 years, ≥35 years), prepregnancy BMI (<25 kg/m^2^, ≥25 kg/m^2^), pregnancy weight gain ratio (<20%, ≥20%), and infant weight (<4000 g, ≥4000 g). Second, it is difficult to match the progress of labor and dilatation of the cervix in pregnant women. We analyzed the association of sEMG with the timing of CD, excluding intrapartum CD when the cervix was dilated to <6 cm.

Statistical analyses were performed using SPSS version 26.0 and R version 4.1.3. All statistical analyses were two‐sided, and statistical significance was set at *p* < 0.05.

## 4 RESULTS

### 4.1 Characteristics of the participants

**FIGURE 1:**
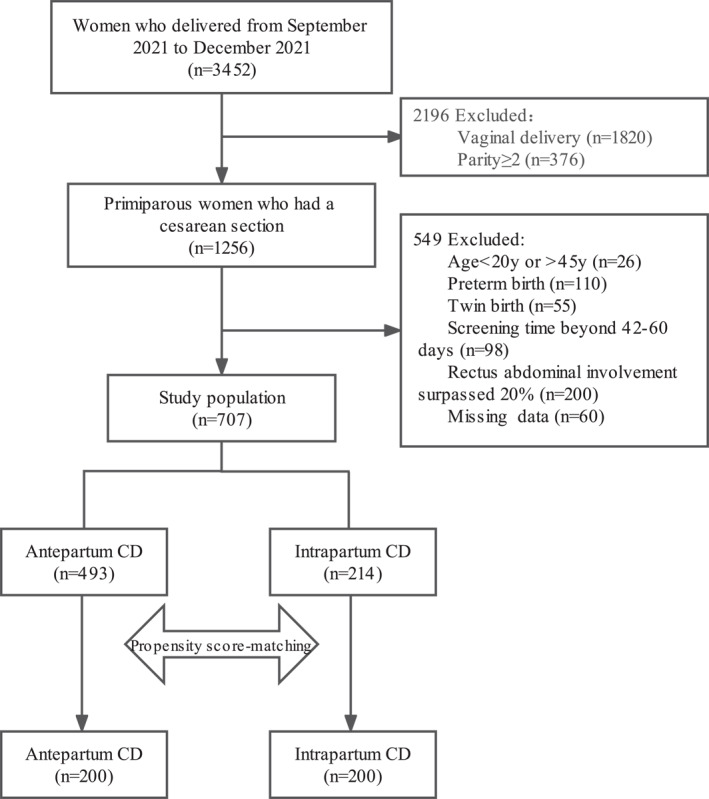
Flow chart for identification of eligible study population. CD, cesarean delivery. Women who failed to attend postpartum screening were included in the missing data.

A total of 3452 women delivered between September 2021 and December 2021. Of these, 707 participants were enrolled in the study after applying the inclusion and exclusion criteria, of which 493 had antepartum CD, and 214 had intrapartum CD. The propensity score‐matched cohort included 400 primiparous women who underwent CD, 200 each in the antepartum and intrapartum CD groups (Figure 1).

The overall population mean (standard deviation [SD]) age was 31.67 (3.14) years, mean (SD) prepregnancy BMI was 21.79 (3.07) kg/m^2^, and median (IQR) gestation week was 39 (38–39) weeks. The differences in partial baseline characteristics before propensity score matching were significant. Women who underwent intrapartum CD had lower age (31.33 [3.07] vs. 31.98 [3.74], *p* = 0.027), higher infant weight (3384.88 g [415.91] vs. 3257.0 g [514.9], *p* = 0.001), higher gestational week (39 [38–40] vs. 39 [38–40], *p* < 0.001), and lower proportion of hypertensive disorders (19 [8.9] vs. 83 [16.8], *p* = 0.008), compared to women who underwent antepartum CD. After propensity matching, 200 individual pairs of study participants were matched and successfully entered into the final cohort, with well‐balanced baseline data (SMD less than 0.1) (Table 1).

### 4.2 Epidural anesthesia with sEMG of PFM and urinary incontinence

In the propensity‐matched cohort, there were no statistically significant differences in sEMG of PFM between women with antepartum CD or intrapartum CD (*p* > 0.05 for all). Transforming each sEMG value into a binary variable according to the median, we found that intrapartum CD did not exhibit a higher risk of adverse outcomes than antepartum CD: high‐level pretest resting baseline (102 [51.0] vs. 98 [49.0]; aOR, 1.09 [95% CI 0.74–1.62]; *p* = 0.658); low‐level fast contractions (95 [47.5] vs. 105 [52.5]; aOR, 0.83 [95% CI 0.56–1.22]; *p* = 00.342); low‐level sustained contractions (97 [48.5] vs. 103 [51.5]; aOR, 0.90 [95% CI 0.61–1.34]; *p* = 0.605); and high‐level posttest resting baseline (107 [53.5] vs. 0.93 [46.5]; aOR, 1.33 [95% CI 0.90–1.99]; *p* = 0.153). Similarly, when using postpartum urinary incontinence (24 [12.0] vs. 21 [10.5]; aOR, 1.12 [95% CI 0.60–2.12]; *p* = 0.717) and stress urinary incontinence (12 [6.0] vs. 14 [7.0]; aOR, 0.80 [95% CI 0.35–1.80]; *p* = 0.596) as study outcomes, we observed similar results (Table 2).

**TABLE 2:** Comparison of surface electromyography (sEMG) values (continuous variables) in the propensity score‐matched cohort with different timing of CD

Parameter of sEMG	Total	Mode of delivery		
		Antepartum CD (*n* = 200)	Intrapartum CD (*n* = 200)	*p* value
Pretest resting baseline, median [IQR], µV	6.1 [3.3–9.0]	6.0 [3.3–8.8]	6.2 [3.3–9.6]	0.539
Fast contractions, median [IQR], µV	45.0 [31.6–56.6]	43.8 [30.3–55.5]	45.6 [34.8–57.4]	0.152
Sustained contractions, median [IQR], µV	31.1 [21.7–39.7]	30.77 [19.94–38.38]	31.94 [22.61–40.20]	0.108
Posttest resting baseline, median [IQR], µV	6.3 [3.9–9.3]	6.1 [3.9–8.7]	6.7 [3.7–9.7]	0.175

Abbreviations: CD, cesarean delivery; IQR, interquartile range; sEMG, surface electromyography.

^a^Wilcoxon test.

### 4.3 Sensitivity analysis

**FIGURE 2:**
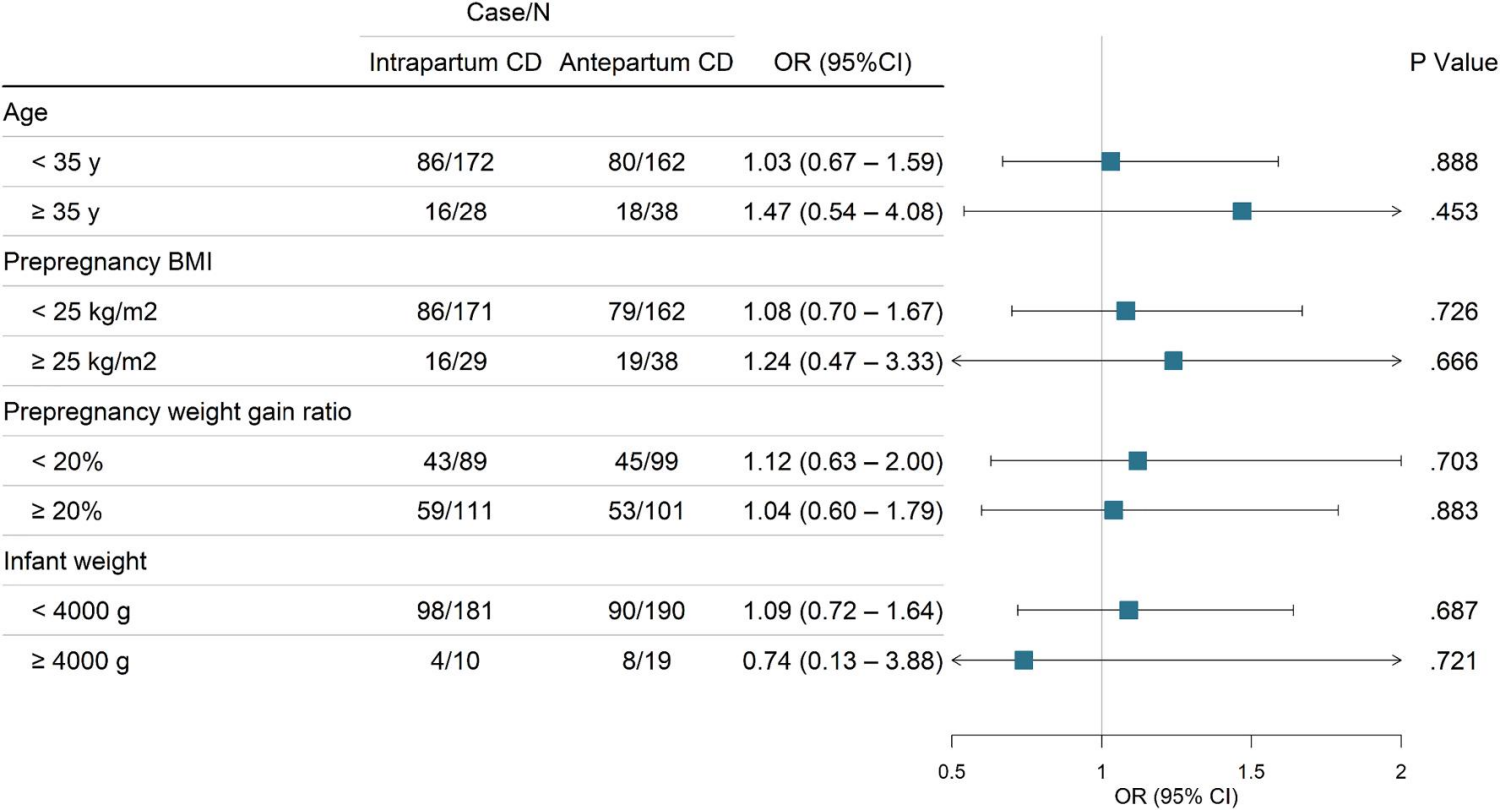
Association of high‐level pretest resting baseline surface electromyography (sEMG) with the timing of CD in subgroup analyses. CD, cesarean delivery; OR, odds ratio. OR and 95% CI were adjusted for screening time after delivery and abdominal involvement ratio.

**FIGURE 3:**
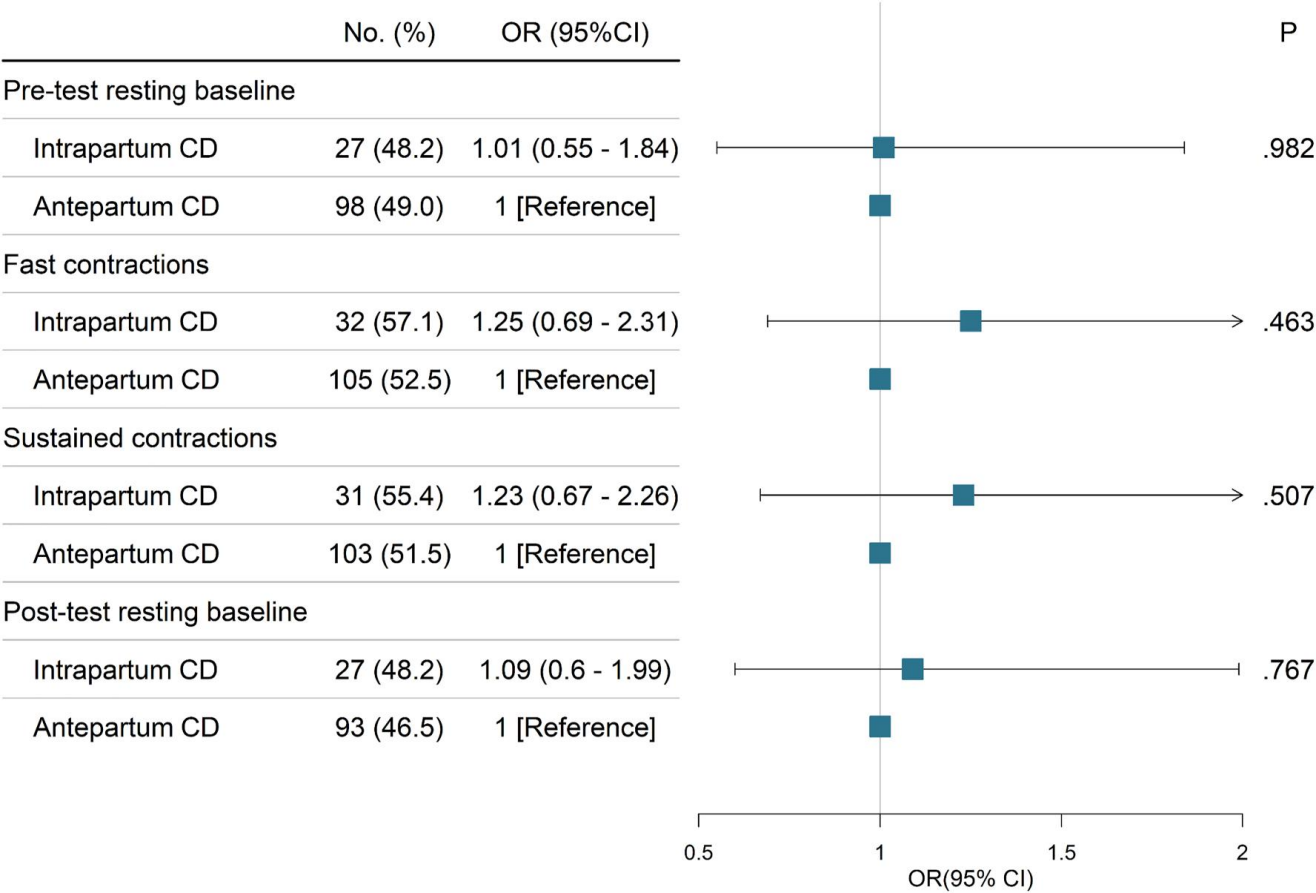
Association of surface electromyography (sEMG) with the timing of cesarean delivery (CD) excluding participants' intrapartum CD when the cervix was dilated less than 6 cm. CD, cesarean delivery; OR, odds ratio. OR and 95% CI were adjusted for screening time after delivery and abdominal involvement ratio.

The results of the stratified analysis based on age, prepregnancy BMI, pregnancy weight gain ratio, and infant weight were identical to the primary findings, with the risk remaining substantially unchanged (Figure 2; Table 1 in the Supplement). We reanalyzed our data after excluding participants with intrapartum CD when the cervix was dilated to <6 cm. All sEMG of PFM had a comparable level of risk as overall participants in the antepartum CD group or intrapartum CD group (Figure 3).

## 5 DISCUSSION

In our study, no statistically significant difference was observed at 42–60 days after delivery in the intrapartum and antepartum CD groups regarding sEMG amplitude and incidence of urinary incontinence. When excluding participants who underwent intrapartum CD when the cervix was dilated to <6 cm, there was still no significant difference in sEMG amplitude between the two groups. The results did not demonstrate the superiority of any of the two modes of delivery in terms of the bioelectrical activity of the PFM.

Although pelvic floor dysfunction has a very high disease incidence, many women often show no clinical symptoms in the early postpartum period. sEMG, noninvasive and reliable, is widely used to identify muscle activity.^16^ It has been reported that the strong correlation between overall muscle strength and the number of activated motor units could be represented by sEMG activity.^17^ The International Urogynecological Association (IUGA) and International Continence Society joint report noted that sEMG could evaluate PFM tone and help patients consciously activate the PFM.^18^ Previous studies confirm that sEMG is interconnected with digital palpation, which is widely used to validate data in research and clinical practice, and that sEMG of the PFM is inversely correlated with age, parity, and incidence of stress urinary incontinence.^16^, ^19^ The resting baseline sEMG reflects the resting activity of the PFM, which play a crucial role in sustaining the proper anatomical position of the pelvic organs. Guo et al. found that women who underwent different delivery modes had different resting baseline sEMG values in the early postpartum period (6–8 weeks after childbirth).^20^ Furthermore, through binary logistic regression analysis, they found that the mode of delivery was a unique factor affecting the pretest resting baseline sEMG. Therefore, sEMG is considered as a method for pelvic floor muscle evaluation in the early postpartum period.

The effect of delivery on pelvic floor dysfunction has received increasing attention over the past few years. The mode of delivery is a significant independent risk factor for pelvic organ prolapse. A cross‐sectional study found that urinary incontinence and pelvic organ prolapse were more prevalent in women who delivered vaginally compared to cesarean section.^21^ A cohort study of women 5–10 years after the first delivery found that severe stress urinary incontinence was more common after vaginal delivery than after CD.^11^ These studies have confirmed that pelvic floor dysfunction is much more common in vaginal delivery than in CD. In addition, operative vaginal delivery was considered an important risk factor for pelvic floor dysfunction, and seems to be associated with a higher prevalence of early pelvic organ prolapse compared to spontaneous vaginal delivery.^22^

On ultrasound, vaginal delivery has been associated with an increased hiatal area, and these changes are more likely to persist after vaginal delivery than after CD.^23^ Similarly, the pretest resting baseline sEMG value in women who underwent elective CD is dramatically higher than in women who experienced spontaneous vaginal delivery.^20^ It is generally believed that vaginal delivery can damage the levator ani muscle, perineal nerve, ligaments, and other supporting pelvic tissues. When pelvic floor support systems are damaged as described, the result is pelvic floor dysfunction.

However, there is little research on the different effects of intrapartum and antepartum CDs on pelvic floor dysfunction. Because of various unique situations, some women would undergo intrapartum CD in the current clinical practice. A cluster‐randomized controlled trial found that approximately 5.9%–9.5% of women in tests of vaginal delivery eventually experience intrapartum CD when adhering to the WHO partograph, and the ratio of intrapartum vaginal delivery can increase with the aging trend of puerpera.^24^ Some think it will damage the soft birth canal during the trial of vaginal delivery and thereby increase the risk of pelvic floor dysfunction. To test this hypothesis, we compared the sEMG of women for different delivery modes at 42–60 days after delivery and found no significant differences between the groups. Unexpectedly, the results indicated that intrapartum vaginal delivery might not cause a change in PFM tone compared with antepartum CD. The outcomes of postpartum urinary incontinence also showed no significant differences between the two groups. Furthermore, this proved that different opportunities for CD might not have other influences on the PFM.

According to the definitions and recommendations for the management of labor dystocia and arrest, which were revised by the American College of Obstetricians and Gynecologists (ACOG) and the Society for Maternal‐Fetal Medicine, the first stage of labor includes the latent phase of labor and active phase of labor, and the active stage of labor does not begin before 6 cm of cervical dilatation is achieved.^25^ In a post hoc analysis of this study, women who underwent intrapartum CD when the cervix was dilated to <6 cm were excluded to eliminate the intervention of women who underwent intrapartum CD during the latent phase of labor. The results showed that the difference in sEMG was still not statistically significant between the two groups. We speculate that injury may be slight or reversible during the first stage of labor, while the pelvic floor muscles undergo huge deformation during the second stage of labor. The birth process was modeled with the help of a three‐dimensional model and the female pelvic MRI data, and it was found that stress peak of levator ani appears when the fetal head moved along the Carus curve to about three‐quarters of the total distance.^26^ A strong relationship between levator ani muscle injury and the duration of the second stage of labor has been emphasized.^27^ These studies partially support our hypotheses. In addition, cesarean delivery is associated with a greater risk of abnormal placentation, uterine rupture, ectopic pregnancy, and preterm birth.^28^ In summary, we consider that the first stage of labor will not lead to serious injury of the pelvic floor muscles and primiparas could be given ample opportunity to try labor.

Our study is the first to report PFM activity in two different modes of CD by directly measuring sEMG in the early postpartum period. We used propensity score matching to correct selection bias due to nonrandom treatment allocation. Although propensity score matching can balance most variables simultaneously, it can only be limited to known confounding variables. Some unknown confounding variables may have affected our results. Second, we only measured PFM sEMG and the incidence of urinary incontinence in the early postpartum period because of limited data. It is not clear whether the two modes of CD have the same influence on the incidence of pelvic floor dysfunction 5 years or longer after childbirth. Further studies are required to confirm the existence of this correlation. Table 3.

**TABLE 3:** Binary logistic regression analysis between surface electromyography (sEMG) level, the incidence of urinary incontinence (UI), and timing of cesarean delivery (CD) in the propensity score‐matched cohort

	Event, No. (%)	OR (95% CI)	*p* value	Adjusted OR (95% CI)^a^	*p* value
Primary outcomes					
Pretest resting baseline^b^					
Intrapartum CD	102 (51.0)	1.08 (0.73–1.60)	0.689	1.09 (0.74–1.62)	0.658
Antepartum CD	98 (49.0)	1 [Reference]		1 [Reference]	
Fast contractions^c^					
Intrapartum CD	95 (47.5)	0.82 (0.55–1.21)	0.318	0.83 (0.56–1.22)	0.342
Antepartum CD	105 (52.5)	1 [Reference]		1 [Reference]	
Sustained contractions^c^					
Intrapartum CD	97 (48.5)	0.89 (0.60–1.31)	0.549	0.90 (0.61–1.34)	0.605
Antepartum CD	103 (51.5)	1 [Reference]		1 [Reference]	
Posttest resting baseline^b^					
Intrapartum CD	107 (53.5)	1.32 (0.89–1.96)	0.162	1.33 (0.90–1.99)	0.153
Antepartum CD	93 (46.5)	1 [Reference]		1 [Reference]	
Second outcomes					
UI					
Intrapartum CD	24 (12.0)	1.16 (0.62–2.18)	0.635	1.12 (0.60–2.12)	0.717
Antepartum CD	21 (10.5)	1 [Reference]		1 [Reference]	
SUI					
Intrapartum CD	12 (6.0)	0.84 (0.38–1.88)	0.685	0.80 (0.35–1.80)	0.596
Antepartum CD	14 (7.0)	1 [Reference]		1 [Reference]	

Abbreviations: CD, cesarean delivery; OR, odds ratio; SUI, stress urinary incontinence; UI, urinary incontinence.

^a^ Adjusted for screening time after delivery and abdominal involvement ratio.

^b^ More than the median as an adverse outcome.

^c^ Less than the median as an adverse outcome.

## AUTHOR CONTRIBUTIONS

Yiyao Chen: Conceptualization, Methodology and Writing ‐ Original Draft. Chuangchuang Xu: Formal analysis and Visualization. Qimanguli Saiding: Writing ‐ Reviewing and Editing. Xiaolei Chi: Data Curation. Lei Chu and Xianjing Wang: Project administration. Xinliang Chen: Funding acquisition and Conceptualization.

## CONFLICT OF INTEREST

The authors declare no conflicts of interest.

## ETHICS STATEMENT

The collection of study population data was approved by the International Peace Maternity and Child Health Hospital (IPMCH) Ethics Committee, and the requirement for informed consent was waived (GKLW 2016‐55). As our study was a retrospective cohort study, clinical trial registration was not required. All procedures were performed in accordance with the ethical standards in the Declaration of Helsinki.

## Supporting information

Supporting Information S1

## Data Availability

Data supporting this study's findings are available from the corresponding author upon reasonable request.
