# Supplementary material for: Association of cesarean delivery timing with pelvic floor muscle function and urine incontinence: A propensity score‐matched study
Source: Smart Med. 2022 Dec 27;1(1):e20220018. doi: 10.1002/SMMD.20220018 (PMC11235817; doi:10.1002/SMMD.20220018)
Supplement: Supplementary file 1 — Supporting Information S1 [file SMMD-1-e20220018-s001.docx]

Supplemental Online Content

**Association of Cesarean Delivery Timing With Pelvic Floor Muscle Function and Urine Incontinence: A Propensity Score Matched Study**

eAppendix 1. Information about pelvic floor muscle (PFM) surface electromyographic (sEMG) value measurement.

eFigure 1. sEMG phase of measurement.

eFigure 2. A) Vaginal probe; B) Patch electrode; C) Biological feedback and stimulation system

eTable 1. Association of low level fast contractions, low level sustained contractions, and high level post-test resting baseline with the timing of cesarean delivery in subgroup analyses.

eFingure 3. Distribution of propensity scores before and after propensity scoring matching.

eAppendix 1. Information about pelvic floor muscle (PFM) surface electromyographic (sEMG) value measurement

The evaluation instrument was CE (CONFORMITE EUROPEENNE) certified, with the following main components: bioelectrical activity receiving device-vaginal probe with metal (CACB04, Med Lander Medical Instruments Co., Ltd., Nanjing, China); signal processing and result output device-Biological Feedback and Stimulation System (MLD B4Plus, Med Lander Medical Instruments Co., Ltd., Nanjing, China). Set the device acquisition frequency range from 0.5 Hz to 1000 Hz, and output the results in microvolts (µV). The testers were trained by both the instrument manufacturer and doctors and were only authorized to operate after passing the test.

Participants were instructed to empty their bladders prior to measurement and to lie in a supine lithotomy position. Vaginal palpation was used to instruct participants on how to apply the correct force to the pelvic floor muscle groups (if the examiner feels inward pressure and/or upward traction when placing their finger within the participant's vagina, the contraction is considered correct). Then a lubricated vaginal probe was gently inserted into the participant's vagina, while patch electrodes were placed on the surface skin of the rectus abdominal muscle simultaneously. Throughout the assessment, voice and visualization screens guided the procedure, which was as follows: "pre-test resting baseline" (a 10-second preliminary resting baseline was measured to determine the PFM's lower basal activity, with the mean value used as the measurement result); "maximum voluntary contraction, MVC" (five rapid contractions, each followed by a 5-second rest period, with the maximum value used as the measurement result); "sustained contraction" (five repetitions of 10-second contractions, each contraction was followed by a 10-second rest period, with the mean value used as the measurement result); "post-test resting baseline"( a 10-second resting baseline was measured to determine the PFM's lower basal activity after movement, with the mean value used as the measurement result). SEMG values of the rectus abdominal were measured simultaneously with PFM measurements, and rectus abdominal involvement was calculated.


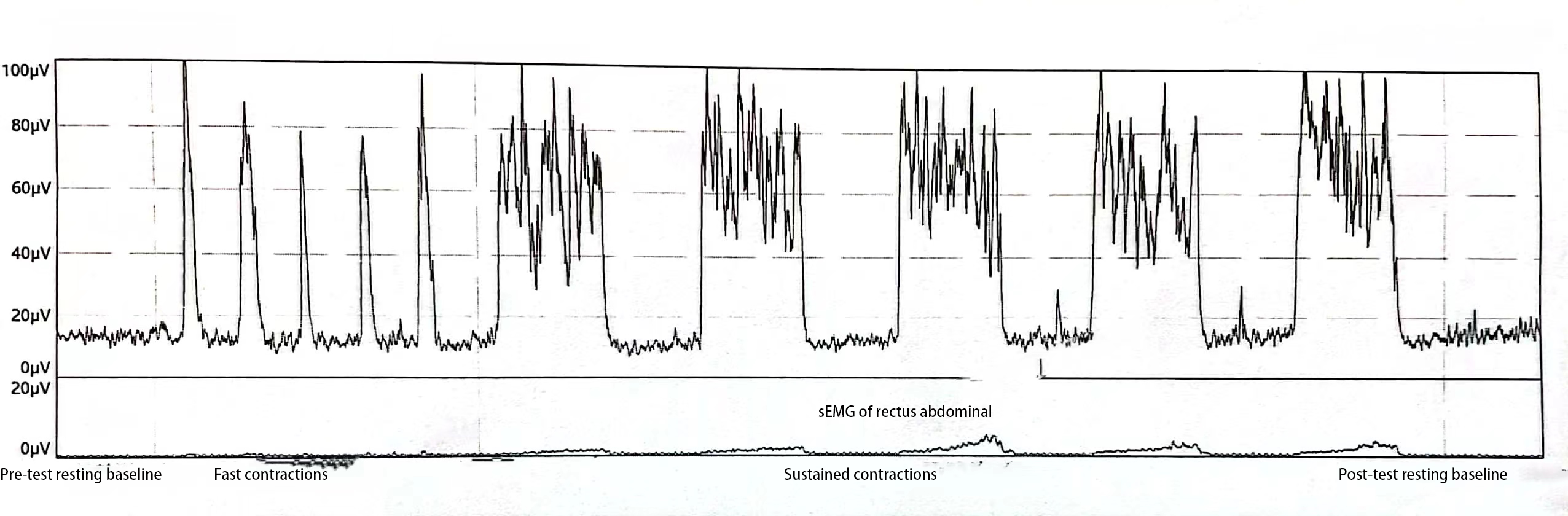


eFigure 1. sEMG phase of measurement


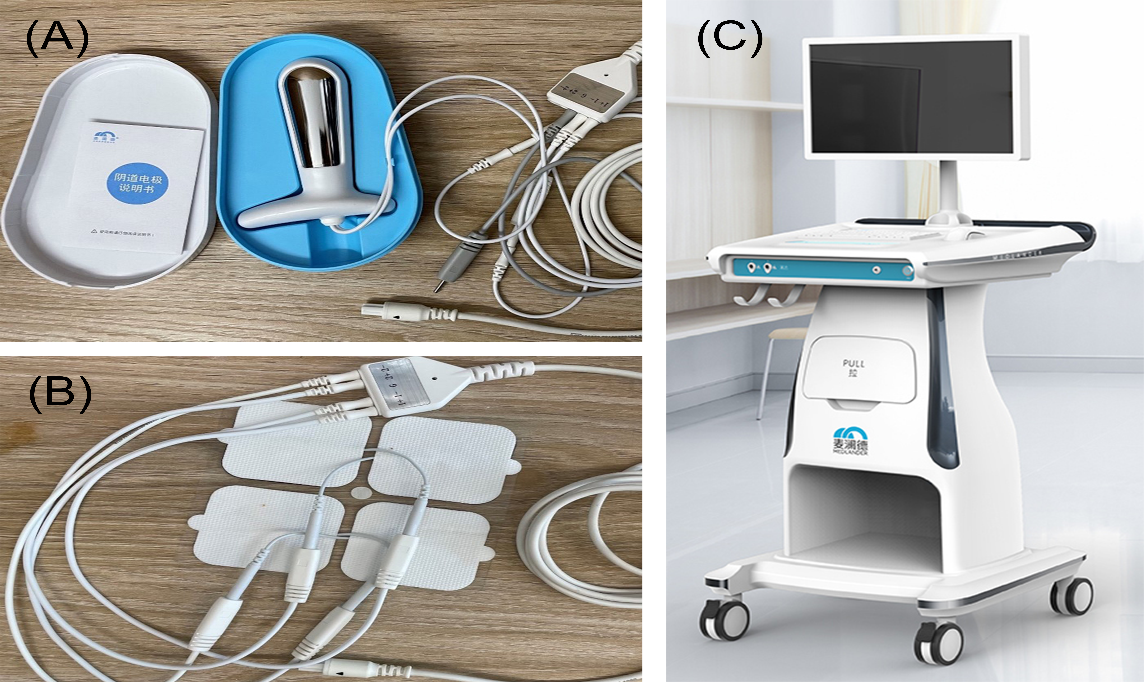


eFigure 2. A) Vaginal probe; B) Patch electrode; C) Biological feedback

and stimulation system

eTable 1. Association of low level fast contractions, low level sustained contractions, and high level post-test resting baseline with the timing of cesarean delivery in subgroup analyses

|  | Age | |  | Preprengnancy BMI | |
| --- | --- | --- | --- | --- | --- |
|  | ＜35 y | ≥ 35 y |  | ＜ 25 kg/m2 | ≥ 25 kg/m2 |
| Fast contractions^a^ | | | | | |
| Intrapartum CD | 0.76 (0.28 – 2.02 ) | 0.84 (0.54 – 1.29 ) |  | 0.43 (0.14 – 1.24 ) | 0.88 (0.57 – 1.36 ) |
| Antepartum CD | 1 [Reference] | 1 [Reference] |  | 1 [Reference] | 1 [Reference] |
| Sustained contractions^b^ | | | | | |
| Intrapartum CD | 0.40 (0.13 – 1.12 ) | 1.02 (0.66 – 1.58 ) |  | 0.78 (0.27 – 2.18 ) | 0.90 (0.58 – 1.39 ) |
| Antepartum CD | 1 [Reference] | 1 [Reference] |  | 1 [Reference] | 1 [Reference] |
| Post-test resting baseline^c^ | | | | | |
| Intrapartum CD | 1.10 (0.40 – 3.00 ) | 1.40 (0.97 – 1.10 ) |  | 1.53 (0.58 – 4.16 ) | 1.30 (0.85 – 2.02 ) |
| Antepartum CD | 1 [Reference] | 1 [Reference] |  | 1 [Reference] | 1 [Reference] |
|  |  | |  |  | |
|  | Weight gain in pregnancy ratio | |  | Infant weight | |
|  | ＜20% | ≥ 20% |  | ＜4000 g | ≥ 4000 g |
| Fast contractions^a^ |  |  |  |  |  |
| Intrapartum CD | 0.80 (0.46 – 1.37 ) | 0.84 (0.47 – 1.50 ) |  | 0.22 (0.03 – 1.15 ) | 0.89 (0.59 – 1.34 ) |
| Antepartum CD | 1 [Reference] | 1 [Reference] |  | 1 [Reference] | 1 [Reference] |
| Sustained contractions^b^ | | | | | |
| Intrapartum CD | 0.73 (0.42 – 1.27 ) | 1.11 (0.63 – 1.99 ) |  | 0.36 (0.06 – 1.85 ) | 0.98 (0.65 – 1.48 ) |
| Antepartum CD | 1 [Reference] | 1 [Reference] |  | 1 [Reference] | 1 [Reference] |
| Post-test resting baseline^c^ | | | | | |
| Intrapartum CD | 1.61 (0.93 – 2.80 ) | 1.07 (0.60 – 1.91 ) |  | 3.50 (0.62 – 4.71 ) | 1.26 (0.84 – 1.90 ) |
| Antepartum CD | 1 [Reference] | 1 [Reference] |  | 1 [Reference] | 1 [Reference] |

eFigure 3. Distribution of propensity scores before and after propensity scoring matching


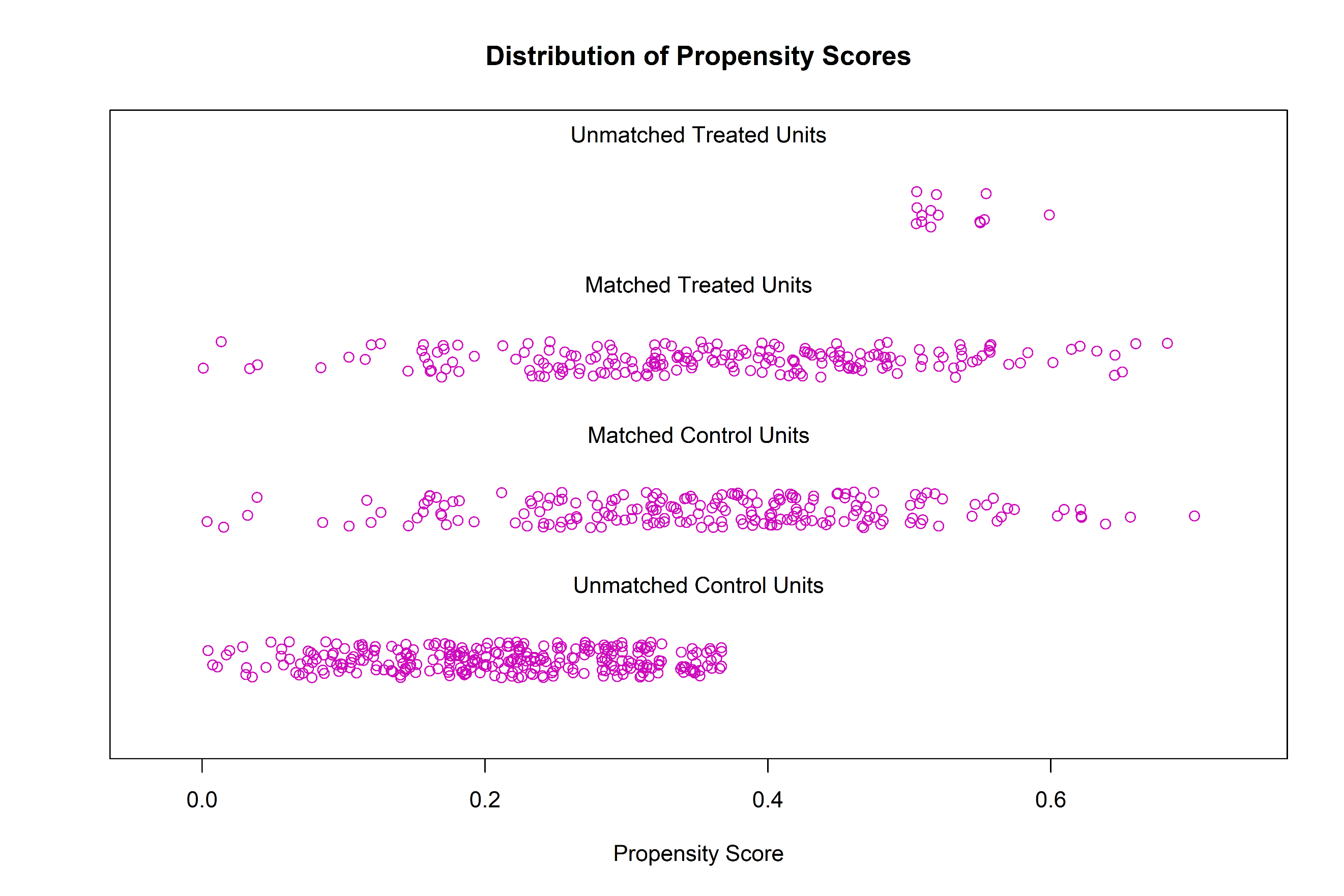


Treatment Units indicates Intrapartum CD group; Control Units indicates Antepartum CD group.
